# A Rare Case of Juvenile Systemic Sclerosis With Concurrent Disseminated Tuberculosis: Diagnostic and Therapeutic Challenges

**DOI:** 10.7759/cureus.75136

**Published:** 2024-12-05

**Authors:** Upendra Prasad Sahu, Omar Hasan, Yuthika Kumari, Naghma Mobin, Mani Shankar

**Affiliations:** 1 Department of Pediatrics, Rajendra Institute of Medical Sciences, Ranchi, IND

**Keywords:** anti-scl-70 antibodies, immunosuppression, juvenilee systemic sclerosis, multidisciplinary management, pediatric autoimmune diseases, pulmonary tuberculosis, raynaud’s phenomenon, tuberculous lymphadenitis

## Abstract

We present the case of a 13-year-old female diagnosed with juvenile systemic sclerosis, diffuse cutaneous subtype, along with active disseminated tuberculosis. This co-occurrence poses unique diagnostic and therapeutic challenges, particularly given the risk of tuberculosis exacerbation due to immunosuppressive therapy required for systemic sclerosis. The patient had signs/symptoms like progressive skin tightening and Raynaud’s phenomenon; the diagnosis was confirmed by the presence of anti-Scl-70 antibodies. Concurrently, active disseminated tuberculosis was identified by a cartridge-based nucleic acid amplification test (CBNAAT) and supported by high-resolution computed tomography (HRCT) thorax and fine needle aspiration cytology (FNAC) of the submandibular lymph node. Treatment involved anti-tuberculosis therapy prior to initiating immunosuppression, ensuring a careful balance between managing autoimmunity and infection. The case emphasizes the importance of multidisciplinary collaboration and vigilant follow-up in managing complex autoimmune conditions coexisting with infectious diseases. Early diagnosis and an individualized approach were crucial to achieving clinical improvement in this adolescent pediatric patient.

## Introduction

Systemic sclerosis (SSc) is a rare autoimmune connective tissue disease characterized by excessive fibrosis affecting the skin and internal organs [1]. The diffuse cutaneous subtype is notably severe, often leading to significant morbidity due to rapid disease progression and multi-organ involvement [2-4]. Pediatric adolescent cases of SSc are rare, presenting challenges in early diagnosis and effective management due to their low incidence rates of between 0.27 and 2.9 cases per million children annually [5-7]. Common clinical features include Raynaud's phenomenon, progressive skin tightening, and the presence of specific autoantibodies [8-9] such as anti-Scl-70.

Understanding the complexities of concurrent tuberculosis

The management of systemic sclerosis becomes even more complicated when it coexists with infections like tuberculosis (TB), particularly in countries with high TB prevalence [10], such as India. Immunosuppressive therapies, essential for controlling SSc, can inadvertently worsen latent or active TB infections [11]. This case report discusses a 13-year-old female diagnosed with juvenile systemic sclerosis alongside active disseminated tuberculosis. It highlights the critical importance of a multidisciplinary approach to balance immunosuppression with effective anti-tuberculosis treatment, aiming to improve patient outcomes while minimizing the risks associated with immunosuppressive therapy.

## Case presentation

A 13-year-old girl presented with pain in her distal fingers, progressive skin tightening, and difficulty with joint movements. She also reported episodes of Raynaud's phenomenon, experiencing cyanosis in her fingertips upon exposure to cold. These symptoms began three years earlier, initially with pain in the distal fingers that gradually worsened. 

Physical examination findings

**Figure 1:**
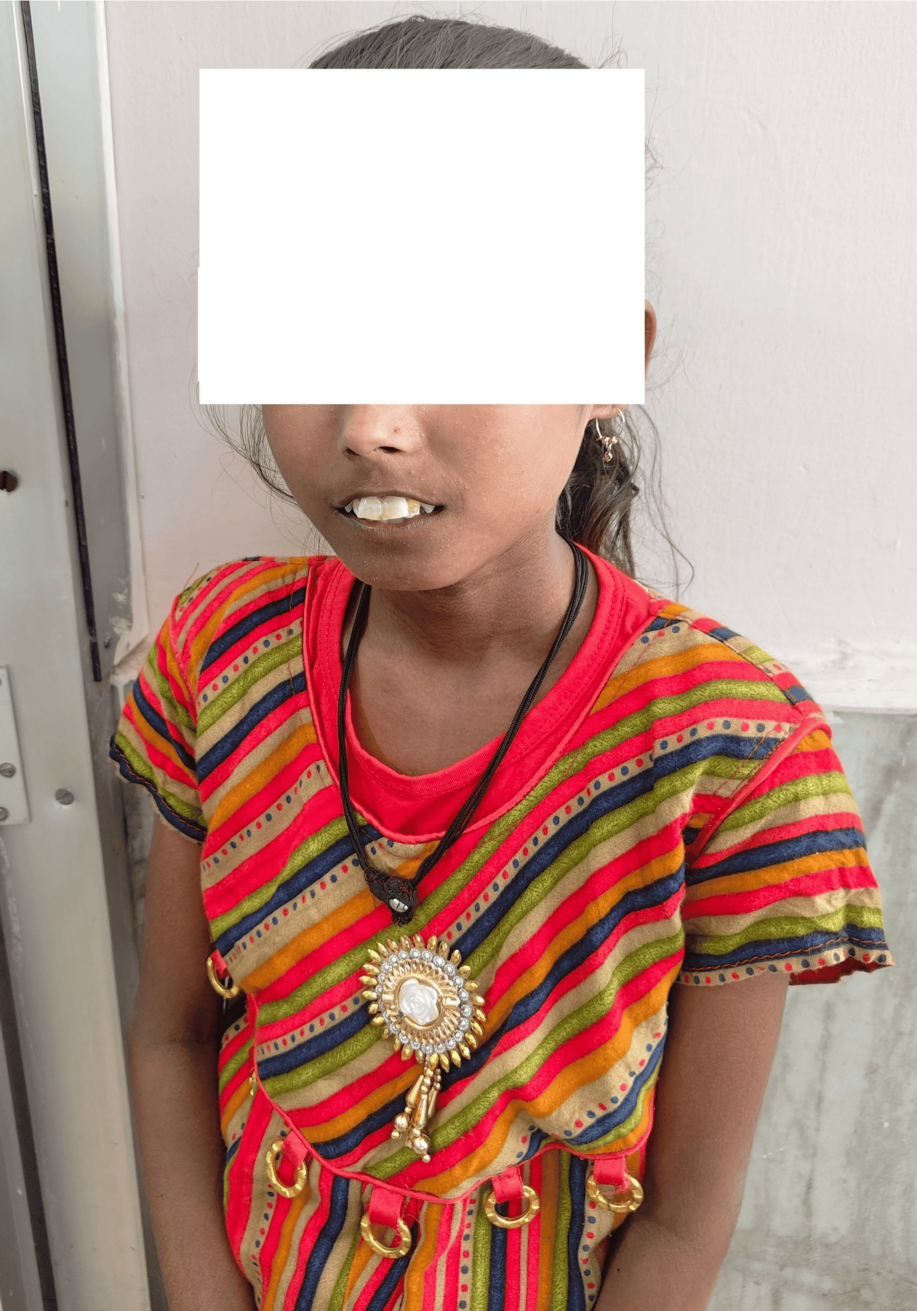
“Fish mouth” appearance

On examination, body mass index (BMI) was 14 kg/m² (<-2 standard deviation), which is classified as thinness according to the World Health Organization (WHO) BMI-for-age criteria. Pallor, cyanosis at the fingertips, grade II clubbing of all fingers, and multiple palpable lymph nodes (mandibular, submental, and supraclavicular) were present, which were significant, with the maximum size of submandibular lymph nodes being 2.5 x 2 cm. Additionally, perioral skin tightening caused a "fish mouth" appearance (Figure 1). 

**Figure 2:**
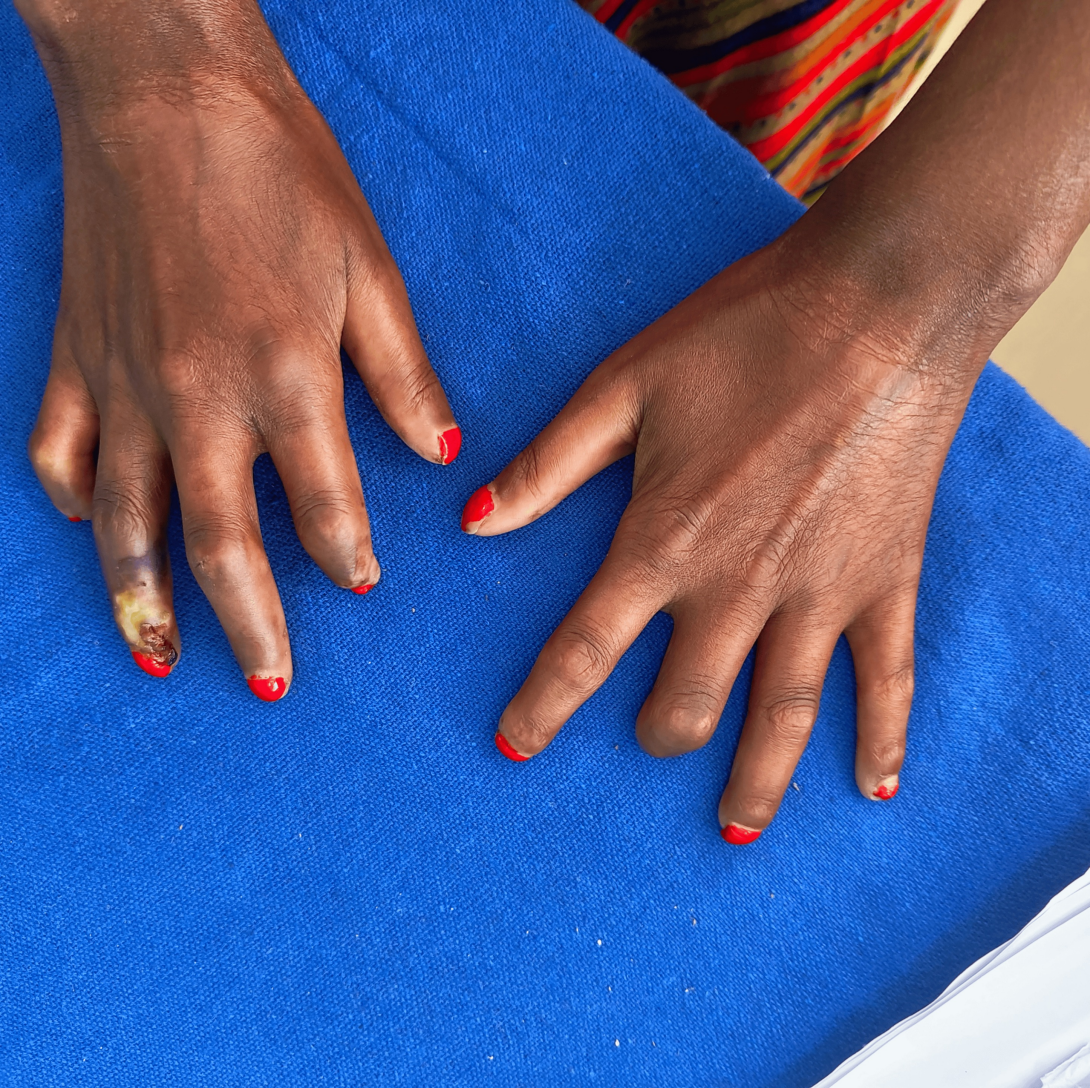
Generalized skin tightening with ulceration of the right ring finger with amputation of the distal middle finger of the left hand

Vital signs were within normal limits. The skin assessment revealed tightness over the fingers (Figure 2), toes, mouth, and extensor and flexor surfaces of the arms.

**Figure 3:**
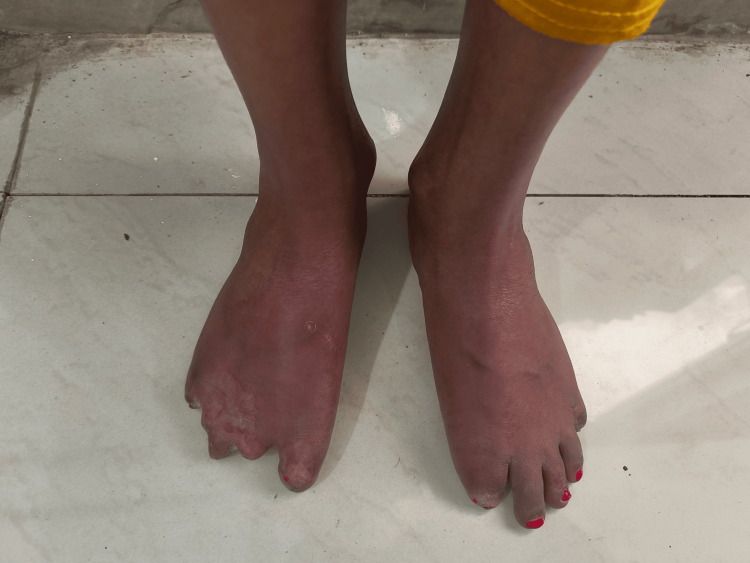
Amputation of multiple toes

Musculoskeletal examination showed limited joint mobility, gangrene, ulceration, and auto-amputation of fingers and toes (Figure 3).

On chest examination, it was revealed that the air entry was diminished on the right upper lung field compared to the left side; bronchial breath sounds and crepitations were heard in the bilateral upper lung field.

Laboratory and imaging studies

**Figure 4:**
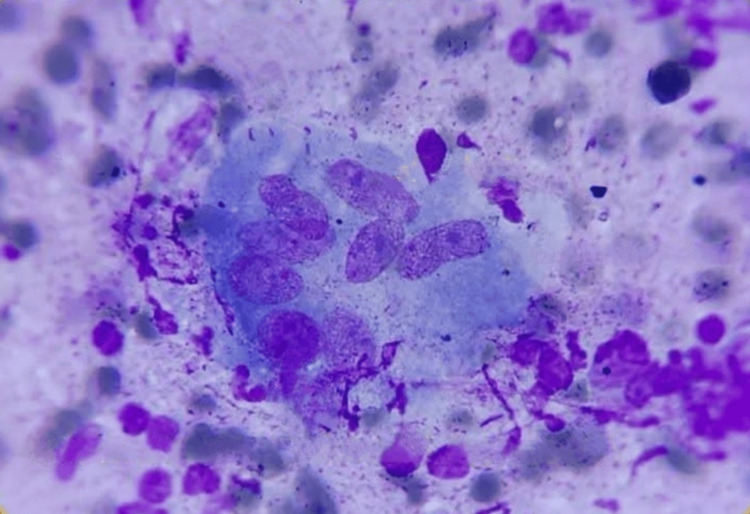
FNAC of lymph nodes The image shows a multinucleated giant cell with a necrotic background and lymphocytes suggestive of a necrotizing granulomatous lesion FNAC: fine-needle aspiration cytology

Laboratory tests (Table 1), imaging studies (Table 2), and histopathological examinations (Table 3, Figure 4) were conducted.

**Table 1:** Laboratory tests ESR: erythrocyte sedimentation rate; SGOT: serum glutamic-oxaloacetic transaminase; AST: aspartate aminotransferase; SGPT: serum glutamic pyruvic transaminase; ALT: alanine aminotransferase; CBNAAT: cartridge-based nucleic acid amplification test

Test	Result	Normal Range
Hemoglobin	8.3 g/dL	11.9–14.8 g/dL
White blood cells (WBC)	7.48 × 10³/µL	4.1–10.4 × 10³/µL
Platelets	314,000/µL	176.9–381.3 × 10³/µL
ESR	50 mm/hr	0–10 mm/hr
SGOT (AST)	50 U/L	13–26 U/L
SGPT (ALT)	20 U/L	8–22 U/L
Urea	17 mg/dL	10–40 mg/dL
Creatinine	0.4 mg/dL	0.45–0.81 mg/dL
Antinuclear antibody	Positive	Negative
Anti-Scl-70 antibody	Positive	Negative
CBNAAT (gastric aspirate)	Positive for Mycobacterium tuberculosis	Negative

**Table 2:** Imaging study

Imaging Study	Findings
High-resolution CT (HRCT) thorax	Fibrotic patches with calcific foci and centrilobular nodular opacities in bilateral upper lobes, multiple calcified mediastinal lymph nodes, moderate pericardial effusion
Echocardiogram (ECHO)	Tachycardia, moderate pericardial effusion, normal biventricular function

**Table 3:** Histopathology findings

Procedure	Findings
Fine-needle aspiration (FNA) of the submandibular lymph node	Necrotizing lesion consistent with tuberculous lymphadenitis

Diagnosis and management plan

The patient was diagnosed with diffuse cutaneous systemic sclerosis complicated by active disseminated tuberculosis. Given the risk that immunosuppressive therapy could exacerbate the tuberculosis infection, a multidisciplinary team initiated anti-tuberculosis treatment before starting immunosuppression. The therapeutic protocol (Table 4) consisted of initiating antitubercular therapy with pyridoxine.

**Table 4:** Therapeutic protocol HRZE: isoniazid, rifampin, pyrazinamide, and ethambutol Dosage [12,13]

Medication/Treatment	Dosage	Route
Prednisolone	1 mg/kg/day	Oral
Methotrexate	1mg/kg/weekly	Subcutaneous
Folinic acid	5 mg/daily	Oral
Nifedipine	0.25 mg/kg/dose	Oral
Pyridoxine (vitamin B6)	10 mg/daily	Oral
Paracetamol	500 mg/SOS	Oral
Anti-tuberculosis therapy (ATT) HRZE	As per guidelines	Oral

On the 14th day of antitubercular therapy, we added Prednisolone at 1 mg/kg/day for seven days, and then rapidly tapered it over the next seven days under strict close monitoring. On the 28th day of treatment initiation, immunosuppressive therapy with prednisolone was switched to methotrexate injections given subcutaneously at 1 mg/kg/weekly dosing. Folinic acid tablets were added to mitigate methotrexate toxicity, and nifedipine was initiated to manage Raynaud's phenomenon. Paracetamol tablets for analgesia were also given, and a dermatology consultation was obtained to manage the skin problems.

## Discussion

This case report presents a rare and challenging clinical scenario of a 13-year-old girl with diffuse cutaneous systemic sclerosis co-occurring with active disseminated tuberculosis. The presence of both conditions in such a young patient is unusual and raises important considerations for management. 

Systemic sclerosis, particularly the diffuse cutaneous subtype, is a rare autoimmune disease characterized by excessive collagen deposition and fibrosis in the skin and internal organs [14]. Its co-occurrence with tuberculosis, an infectious disease caused by Mycobacterium tuberculosis, poses a therapeutic dilemma. Immunosuppressive therapy [15], the cornerstone of diffuse cutaneous systemic sclerosis management, can potentially worsen tuberculosis infection, leading to disseminated disease and poor outcomes. 

In this case, the patient presented with classic features of diffuse cutaneous systemic sclerosis, including Raynaud's phenomenon, skin tightening, digital ulcers, and musculoskeletal involvement. The diagnosis was confirmed by positive antinuclear and anti-Scl-70 antibodies [16]. Additionally, the patient had active disseminated tuberculosis, evidenced by a positive cartridge-based nucleic acid amplification test (CBNAAT) [17] test and characteristic findings on high-resolution computed tomography (HRCT) imaging and fine needle aspiration cytology (FNAC) of lymph nodes. 

Echocardiography and HRCT thorax revealed pericardial effusion was likely multifactorial, attributed to disseminated tuberculosis and diffuse cutaneous systemic sclerosis. Tuberculosis may cause effusion through direct pericardial infection or lymphatic spread, while systemic sclerosis may contribute via chronic inflammation and fibrosis.

The management approach in this case required a delicate balance between controlling the autoimmune disease and preventing the progression of tuberculosis. A multidisciplinary team, including rheumatologists, pulmonologists, and infectious disease specialists, collaborated to develop a treatment strategy that prioritized the patient's safety and well-being. 

The initial step involved treating the active tuberculosis infection with a standard anti-tuberculosis regimen. Once the infection was deemed under control, immunosuppressive therapy with prednisolone was cautiously introduced, followed by methotrexate. Close monitoring for any signs of tuberculosis reactivation was crucial throughout the treatment course.

At follow-up, the patient demonstrated significant clinical improvement, with a marked decrease in joint pain and a reduction in the severity of Raynaud's phenomenon. On physical examination, the cardiovascular system (CVS) findings and fundoscopic examination of the eyes were normal. Complete blood count (CBC) showed mild anemia, while renal function tests (RFT) were within normal limits, and other hematological parameters were stable. These findings suggest effective disease management, with continued monitoring required for anemia. 

This case highlights the complexities of managing patients with concurrent autoimmune and infectious diseases. The potential for immunosuppressive therapy to worsen infections needs a careful risk-benefit assessment and individualized treatment strategies. The successful management of this patient underscores the importance of a multidisciplinary approach and close collaboration among specialists.

## Conclusions

This case highlights the intricate interplay between autoimmune diseases and infections, emphasizing the crucial need for individualized, multidisciplinary care in managing rare and complex clinical scenarios. The successful treatment of this patient with concurrent diffuse cutaneous systemic sclerosis and active disseminated tuberculosis demonstrates that with vigilant monitoring and a carefully balanced therapeutic approach, positive outcomes are attainable even in the face of significant medical challenges. Continued research and additional case studies are essential to expand the understanding of these rare co-occurring conditions and to optimize treatment strategies for similar patients.
